# Hsa-miR-7974 Suppresses Epstein-Barr Virus Reactivation by Directly Targeting *BZLF1* and *BRLF1*

**DOI:** 10.3390/v17050594

**Published:** 2025-04-23

**Authors:** Haotian Li, Hui Wang, Jiao Wang, Xuexin Lu, Jieqiong Zhang, Mingming Wang, Dongbo Yu, Ying Li, Shiwen Wang

**Affiliations:** National Key Laboratory of Intelligent Tracking and Forecasting for Infectious Diseases, National Institute for Viral Disease Control and Prevention, Chinese Center for Disease Control and Prevention, Beijing 102206, China; gonzalezlht7@gmail.com (H.L.); nkwh2000@163.com (H.W.); wangjiao@ivdc.chinacdc.cn (J.W.); 15201586121@163.com (X.L.); zhangjq@ivdc.chinacdc.cn (J.Z.); wangmm@ivdc.chinacdc.cn (M.W.); superbo666@gmail.com (D.Y.)

**Keywords:** Epstein-Barr virus, hsa-miR-7974, lytic cycle, reactivation

## Abstract

Epstein-Barr virus (EBV) reactivation, a key factor in Epstein-Barr virus (EBV)-associated malignancies, is regulated by specific cellular microRNAs (miRNAs). This study investigated the role of Hsa-miR-7974 (miR-7974) in this process. miRNA sequencing revealed significant downregulation of miR-7974 in reactivated EBV-positive cell lines (Raji and C666-1). Bioinformatics prediction and dual-luciferase assays confirmed the direct targeting of the EBV immediate-early gene BRLF1 by miR-7974. Furthermore, miR-7974 mimics suppressed, whereas inhibitors increased, the expression of key EBV lytic genes (BZLF1, BRLF1, and BMRF1) and the viral load, as validated by RT-qPCR. Bioinformatics analyses revealed the involvement of miR-7974 in cellular pathways such as membrane dynamics and signal transduction (MAPK, NF-κB, and IL-10), and its association with Hodgkin’s lymphoma, leukemia, and nasopharyngeal neoplasms. These findings establish that miR-7974 functions as a crucial negative regulator of EBV reactivation by directly targeting BRLF1, highlighting its potential significance in the pathogenesis of EBV-associated malignancies.

## 1. Introduction

Epstein-Barr virus (EBV) is a ubiquitous human gammaherpesvirus that establishes lifelong latent infection in more than 90% of the adult population worldwide [1,2]. EBV is considered an oncogenic virus owing to its ability to promote malignant transformation and stimulate tumor growth, and it has been implicated in several lymphoid and epithelial cancers including Burkitt’s lymphoma [3], Hodgkin’s lymphoma [4], post-transplant lymphoproliferative disorder [5], nasopharyngeal carcinoma [6], and gastric carcinoma [7]. Following primary infection, EBV persists indefinitely in the host through the establishment of latent or lytic cycles of infection [8]. The lytic stage of EBV is essential for horizontal viral spread and the maintenance of persistent infection, but its mechanistic role in viral oncogenesis remains unclear. The switch from EBV latency to lytic replication is widely demonstrated to be modulated by viral and host miRNAs [9].

MicroRNAs (miRNAs) are short and non-coding RNA molecules, typically consisting of 18–24 nucleotides, that play crucial roles in gene regulation. MiRNAs exert gene silencing primarily through post-transcriptional mechanisms [10]. Following their biogenesis, mature miRNAs are incorporated into the RNA-induced silencing complex (RISC), which facilitates the recognition and binding of the miRNA to its target messenger RNA (mRNA). This interaction is predominantly mediated through sequence complementarity between the “seed region” of the miRNA (nucleotides 2–7) and the 3′ untranslated region (3′ UTR) of the target mRNA [11]. Upon binding, miRNAs can elicit gene silencing through two primary mechanisms: translational repression or mRNA degradation [12]. Translational repression involves the inhibition of ribosome recruitment and/or translation initiation, resulting in reduced protein synthesis from the target mRNA [13]. This mechanism is often observed when a miRNA exhibits imperfect complementarity to its target [14]. Alternatively, miRNAs can induce mRNA degradation through the recruitment of deadenylation and decapping enzymes, leading to the rapid decay of the target mRNA [15]. This mechanism typically occurs when the miRNA exhibits extensive complementarity to its target, often resulting in endonucleolytic cleavage of the mRNA [16].

EBV encodes more than 44 mature miRNA sequences that have been experimentally identified [8]. The latent-lytic switch of EBV, which is critically regulated by the interplay of viral and cellular miRNAs, plays a pivotal role in EBV-associated tumorigenesis [17]. During the latent phase, EBV-encoded miRNAs, notably the miR-BARTs, function to repress the expression of viral immediate-early genes, such as *BZLF1* and *BRLF1*, as well as host factors that facilitate lytic reactivation, thereby ensuring the maintenance of viral latency [18,19]. Conversely, cellular miRNAs exert a dualistic influence on the latent-lytic switch, either promoting or inhibiting it through direct targeting of viral transcripts or modulation of host signaling cascades. Hsa-miR-141 has been demonstrated to inhibit *FOXO3* expression, resulting in enhanced EBV lytic reactivation [20]. Hsa-miR-190 targets and downregulates *NR4A3*, a cellular immediate-early gene for EBV reactivation, and inhibits the expression of *BZLF1* and viral lytic DNA replication [21]. Dysregulation of host miRNAs may accelerate the progression of EBV-associated malignancies by modulating key oncogenic pathways or dampening antitumor immune responses [22,23]. However, the specific cellular miRNAs involved in the regulatory roles of the latent-lytic transition remain underexplored.

Hsa-miR-7974 (miR-7974), a mature human miRNA, was first investigated and characterized in 2013 [24]. Its genomic locus is located on chromosome 19, specifically at chr19:11495544–11495622. The functional role of miR-7974 in oncogenesis remains ambiguous. While studies have reported elevated expression levels of miR-7974 in breast cancer [25] and colorectal cancer cells [26], suggesting a potential oncogenic function, contrasting evidence has demonstrated its downregulation in SHG-8- treated glioblastoma samples, which target the oncogenes *CORO2A*, *APC2*, and *WNT7A* [27]. Furthermore, miR-7974 has been reported to be downregulated in glioma tissues, potentially by targeting *BOC*. The downstream genes of *BOC*, *POU2F1*, and *TP53* are implicated in the transcriptional regulation of tumor-related genes [28]. However, it is noteworthy that research on miR-7974 remains relatively limited compared to other well-studied miRNAs. The specific functions and regulatory networks of miR-7974 in EBV reactivation are not yet fully understood.

Through miRNA sequencing, we discovered that miR-7974 was expressed at lower levels in reactivated EBV-positive Burkitt’s lymphoma Raji cells and nasopharyngeal carcinoma C666-1 cells than in control cells, with subsequent validation by quantitative reverse transcription-polymerase chain reaction (RT-qPCR). Functional studies using miR-7974 mimics and inhibitors demonstrated that *BZLF1* and *BRLF1* are direct targets of miR-7974. Furthermore, GO analysis and KEGG pathway analysis were employed to identify biological pathways enriched among the predicted gene targets of the significantly regulated miRNAs, and we also constructed a miRNA–mRNA interaction network of the miR-7974 and EBV lytic genes. These results indicate important roles for miR-7974 in modulating the EBV lytic-latent cycle and contribute to a deeper understanding of the molecular mechanisms underlying EBV reactivation.

## 2. Materials and Methods

### 2.1. Cells and Cell Culture

The EBV-positive human Burkitt’s lymphoma cell line Raji was historically preserved in our laboratory. The human embryonic kidney cell line HEK293T was purchased from Cell Resource Center, Institute of Basic Medical Sciences, CAMS/PUMC (Beijing, China). The EBV-positive human nasopharyngeal carcinoma cell line C666-1 was purchased from Meisen CTCC (Hangzhou, China). The EBV-negative human nasopharyngeal carcinoma cell line HK-1 was purchased from CHI Scientific, Inc. (Shanghai, China). Raji and C666-1 cells were cultured in RPMI 1640 medium (Gibco, Thermo Fisher Scientific, Waltham, MA, USA) supplemented with 10% fetal bovine serum (FBS) (Gibco, Thermo Fisher Scientific, Waltham, MA, USA) and 1% penicillin-streptomycin (Gibco, Thermo Fisher Scientific, Waltham, MA, USA). HEK293T cells were cultured in Dulbecco’s modified Eagle’s medium (DMEM) (Gibco, Thermo Fisher Scientific, Waltham, MA, USA) supplemented with 10% FBS and 1% penicillin-streptomycin. HK-1 cells were cultured in a specialized medium provided by CHI Scientific, Inc., Maynard, MA, USA. All the cell lines were maintained at 37 °C in a humidified atmosphere containing 5% CO_2_.

### 2.2. Epstein-Barr Virus (EBV) Lytic Replication Induction

Raji cells were treated with 20 ng/mL 12-O-tetradecanoylphorbol-13-acetate (TPA; Cell Signaling Technology, Cat# 4174S, Danvers, MA, USA) for 48 h, and C666-1 cells were induced with 40 μmol/L Dp44mT (C7, EBV lytic cycle inducer-1; MedChemExpress, Cat# HY-149577, Monmouth Junction, NJ, USA) for 48 h to initiate EBV lytic replication.

### 2.3. Transcriptome Sequencing

RNA sequencing was performed, and libraries were constructed by Novogene Co., Ltd. (Beijing, China). The reactivated and untreated samples were processed and sequenced on a NovaSeq X Plus Series PE150 platform (Illumina, San Diego, CA, USA). Messenger RNA was purified from total RNA via poly-T oligo-attached magnetic beads. After fragmentation, the first strand cDNA was synthesized via random hexamer primers. Then, the second strand cDNA was subsequently synthesized via dUTP, instead of dTTP. The directional library was ready after end repair, A-tailing, adapter ligation, size selection, USER enzyme digestion, amplification, and purification. The library was checked with a Qubit and real-time PCR for quantification and a bioanalyzer for size distribution detection. After library quality control, different libraries were pooled based on the basis of the effective concentration and targeted data amount, and then subjected to Illumina sequencing.

### 2.4. MicroRNA (miRNA) Deep Sequencing

MiRNA sequencing was performed, and libraries were constructed by Novogene Co., Ltd. (Beijing, China). The reactivated and untreated samples were processed and sequenced on an Illumina SE50 platform (Illumina, San Diego, CA, USA). The 3′ and 5′ adaptors were ligated to the 3′ and 5′ ends of the small RNA, respectively. Then, first-strand cDNA was synthesized after hybridization with reverse transcription primers. The double-stranded cDNA library was generated through PCR enrichment. After purification and size selection, libraries with insertions between 18 and 40 bp were ready for sequencing via Illumina sequencing with SE50. The library was checked with a Qubit and real-time PCR for quantification and a bioanalyzer for size distribution detection. The quantified libraries will be pooled and sequenced on Illumina platforms, according to the effective library concentration and data amount needed.

### 2.5. Differential MicroRNA (miRNA) Expression Analysis

Differential expression analysis was performed via the DESeq2 R package. For regulation calculations between the comparison, a *p*-value < 0.001 and a |log2(FoldChange)| > 1.5 were set as the thresholds for significantly differential expression.

### 2.6. In Silico Prediction of Viral and Cellular Targets of miR-7974

To identify direct targets of miR-7974, the computational prediction tools miRanda (https://github.com/hacktrackgnulinux/miranda, accessed on 5 February 2025) and RNAhybrid (https://bibiserv.cebitec.uni-bielefeld.de/rnahybrid, accessed on 5 February 2025) were utilized to predict potential miRNA binding sites within the EBV genome. For the prediction of potential cellular target genes of miR-7974, the miRDB (https://mirdb.org, accessed on 10 February 2025) and TargetScan (https://www.targetscan.org, accessed on 10 February 2025) databases were used. These platforms integrate sequence-based and context-specific features to predict miRNA–mRNA interactions, providing a comprehensive list of putative cellular targets for further experimental validation.

### 2.7. MiR-7974 Target Gene Enrichment for Biological Pathways, Disease Associations, and Gene Ontology (GO) Categories

To identify pathways and disease associations possibly regulated by miR-7974, the predicted genes were uploaded to the Functional Annotation Tools of DAVID Bioinformatic Resources 6.7 (https://davidbioinformatics.nih.gov). An enrichment was considered significant when the *p*-value was <0.05. Gene Ontology (GO) enrichment analysis and functional visualization were performed via ClueGO 2.5.9 and the CluePedia 1.5.9 plugin in the Cytoscape software 3.10.3.

### 2.8. Establishment of miRNA–mRNA Network

Nine key genes associated with EBV reactivation were first selected to construct the miRNA–mRNA regulatory network, which regulates the key genes involved in EBV reactivation. Cellular miRNAs predicted to target these genes were identified via both RNAhybrid and miRanda tools to analyze sequence complementarity and thermodynamic stability for miRNA–mRNA interactions. The predicted miRNA–mRNA pairs were then imported into Cytoscape, a bioinformatics software platform (3.10.3), to visualize and analyze the regulatory network.

### 2.9. Dual-Luciferase Reporter Assay

To investigate the effect of Hsa-miR-7974 on the expression of EBV immediate-early genes, the 3′ UTR sequences of *BRLF1* and the corresponding mutants were cloned and inserted into the pmirGLO dual-luciferase miRNA target expression vector (Promega, Madison, WI, USA), which were named *BRLF1*-WT and *BRLF1*-MUT. HEK293T cells and HK-1 cells were seeded in a 96-well plate. After 24 h, the cells were co-transfected with the luciferase reporter vector and 20 nM miR-7974 mimics or scrambled miRNA (miR-NC). The relative luciferase activity was measured at 48 h post-transfection via a dual-luciferase reporter gene assay kit (Yeasen, Shanghai, China). For each sample, *Renilla* luciferase activity was normalized to *firefly* luciferase activity.

### 2.10. Stem-Loop Quantitative Real-Time Polymerase Chain Reaction (PCR) for MicroRNA (miRNA) Analysis

MiRNAs were extracted from the cell samples via the miRNeasy Micro Kit (Qiagen, Cat# 217084, Hilden, Germany), following the manufacturer’s protocol. The reverse transcription primers were pre-treated as follows to guarantee a stem-loop structure: they were incubated at gradient temperatures of 95, 80, 70, 60, 50, 40, 30, and 20 °C, for 30 s each. cDNA was synthesized via the PrimeScript RT Reagent Kit with gDNA Eraser (TaKaRa, Cat# RR047A, Kyoto, Japan) according to the manufacturer’s instructions. Before reverse transcription, the mixture was incubated at 16 °C for 15 min to anneal the primers. The sequences of the primers used were as follows: miR-7974 stem-loop RT primer: GTCTGTATGCTTGTTCTCGTCTCTGTGTCATCCCTCAAGCATACAGACGGGCTC, forward primer: TACAGGCTGTGATGCTCTCCT, reverse primer: TATGCTTGTTCTCGTCTCTGTGTC; and snRNA U6 stem-loop RT/reverse primer: AACGCTTCACGAATTTGCGT, forward primer: CTCGCTTCGGCAGCACA. Reverse transcription was carried out in a T100 thermal cycler (Bio-Rad, Hercules, CA, USA) for 15 min at 37 °C, 5 s at 85 °C, and then held at 4 °C. Real-time quantitative PCR was performed via an SYBR Green Premix Pro Taq HS qPCR Kit (Accurate Biotechnology, Guangzhou, China) on an Archimed X6 System (RocGene, Beijing, China). All amplifications were performed in triplicate, and values were normalized to the value for an endogenous control, snRNA U6.

### 2.11. Transfection of MicroRNA (miRNA) Mimics and Inhibitors

The mimic, the inhibitor, and the scrambled control were purchased from Beijing TsingKe Biotech Co., Ltd. (Beijing, China). In this study, C666-1 cells were seeded 24 h prior to transfection in a 6-well-plate containing 2 mL of RPMI 1640 medium. The cells were transfected with negative controls, miR-7974 mimic or miR-7974 inhibitor via the Lipofectamine RNAiMAX (Invitrogen, Cat# 13778030, Thermo Fisher Scientific, Waltham, MA, USA) transfection agent, according to the manufacturer’s protocol. The final concentrations of the miRNA mimic and inhibitor were 10 nM and 50 nM, respectively. After 24 h, the cells were treated with 40 μmol/L C7 compound for 48 h to induce the lytic cycle of EBV.

### 2.12. Quantitative Reverse Transcription-Polymerase Chain Reaction (RT-qPCR)

Total RNA was extracted from the cell samples via an RNeasy Mini Kit (Qiagen, Cat# 74106), following the manufacturer’s protocol. The quality of the extracted RNA was determined on an FC-3100 spectrophotometer (Lifereal Biotechnology, Hangzhou, China). Total RNA reverse transcription was performed via the PrimeScript RT Reagent Kit with gDNA Eraser (TaKaRa, Cat# RR047A, Japan) to generate cDNA. RT-qPCR was carried out via the use of an SYBR Green Premix Pro Taq HS qPCR Kit (Accurate Biotechnology, China) on an Archimed X6 System (RocGene, China). The sequences of the primers used were as follows: *BZLF1* forward: TTGGGCACATCTGCTTCAACAGGA, reverse: AATGCCGGGCCAAGTTTAAGCAAC; *BRLF1* forward: GAACATACCTTCCCGGCTATC, reverse: GAGCGATGAGAGACCCATATTC; *BMRF1* forward: GCCGTTGAGGCCCACGTTGT, reverse: TGGGAATGGCAGGCGAGGGT; and *GAPDH* forward: GACACCCACTCCTCCACCTTT, reverse: ACCACCCTGTTGCTGTAGCC. The PCR conditions were 95 °C for 30 s, followed by 40 cycles at 95 °C for 5 s, 60 °C for 30 s, with fluorescence measured continuously. The relative mRNA expression levels of *BZLF1*, *BRLF1*, and *BMRF1*, normalized to that of *GAPDH* as an internal control, were calculated via the 2^−ΔΔCt^ method.

### 2.13. Epstein-Barr Virus (EBV) DNA Viral Load

Viral-derived DNA was measured to determine the EBV viral load. Briefly, pelleted cells were harvested, and the total DNA was extracted via the QIAamp DNA Mini Kit (Cat# 51304, Qiagen, USA), according to the kit’s handbook. The quality of the extracted RNA was determined on an FC-3100 spectrophotometer (Lifereal Biotechnology, China). To quantify EBV copy numbers, the given standard samples (from 10^7^ copies/µL to 10 copies/µL) were first used to create the standard curve according to the manufacturer’s instructions by using an EBV Probe quantitative PCR Kit (Cat# 15-60800, BINGENE, Beijing, China). Then, 40 ng (2 µL) of each DNA sample was added to PCR tubes, and the PCR was performed as follows (Archimed X6 System, China): 95 °C for 10 min, 95 °C for 15 s, and 60 °C for 60 s (data collection) for 40 cycles. The EBV copy number of each sample can be calculated by the corresponding threshold (Ct) cycle with the aid of a standard curve.

### 2.14. Western Blot Analysis

Total protein was extracted from cell samples lysed in RIPA buffer (Abcam, Cat# ab156034, Waltham, MA, USA) containing a 1% protease inhibitor cocktail. The protein concentration was determined via a BCA assay. Proteins were separated by 10% SDS-PAGE (Beyotime, Shanghai, China) and transferred to 0.45 μM PVDF membranes (Millipore, Billerica, MA, USA) via a semidry transfer system (Bio-Rad, USA). The membranes were blocked with 5% (*w*/*v*) non-fat dried milk in TBST for 1 h at room temperature and then incubated overnight at 4 °C with the following primary antibodies: ZEBRA (1:250; Santa Cruz Biotechnology, Cat# sc-53904, Dallas, TX, USA), Ea-D (1:500; Sigma-Aldrich, Cat# MAB8186, Burlington, MA, USA), and α-Actinin (1:5000; Proteintech, Cat# 11312-2-AP, Wuhan, China). After washing, the membranes were incubated for 1 h at room temperature with HRP-conjugated secondary antibodies (rabbit, 1:20,000, Abcam, Cat# ab205718; mouse, 1:20,000, Abcam, Cat# ab205719). Immunoreactive bands were visualized via enhanced chemiluminescence (Pierce Plus-ECL; Revvity, Waltham, MA, USA) and captured with a CLINX ChemiScope 6200 touch imaging system (Qinxiang, Hangzhou, China). Band intensities were quantified via densitometry via ImageJ software (v1.54 m).

### 2.15. Statistical Analysis

Statistical analyses were performed via R (version 4.4.2) and GraphPad Prism (version 10.3.1). The results are presented as the means ± standard deviations (SDs) of at least three independent experiments, and a two-tailed Student’s *t*-test was used to demonstrate statistical significance. Differences were considered as statistically significant when the *p*-value was <0.05.

## 3. Results

### 3.1. TPA Induces Epstein-Barr Virus (EBV) Lytic Replication in Raji Cells and C7-Induced C666-1 Cells

**Figure 1:**
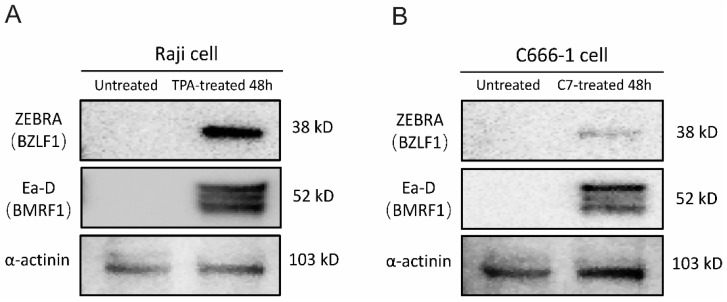
TPA and C7 induce EBV lytic replication in Raji and C666-1 cells. Western blot analysis was used to examine the protein expression of ZEBRA (encoded by the *BZLF1* gene), an immediate-early marker of EBV lytic reactivation, and Ea-D (encoded by the *BMRF1* gene), an early lytic protein. (**A**) Treatment of Raji cells with TPA for 48 h resulted in increased expression of both ZEBRA and Ea-D. (**B**) Incubation of C666-1 cells with C7 for 48 h also resulted in elevated ZEBRA and Ea-D protein expression.

TPA and C7 have been shown to induce lytic reactivation of EBV, respectively. We employed Western blot analysis to assess the expression of viral lytic proteins and mRNA sequencing to examine changes in the host and viral transcriptomes following treatment with TPA or C7. Western blot analysis revealed a significant increase in the protein levels of key EBV lytic cycle markers, including BZLF1 (ZEBRA) and BMRF1 (Ea-D), upon treatment with either TPA or C7 in Raji and C666-1 cells. This upregulation of viral proteins confirmed the successful induction of EBV lytic reactivation in both cell lines (Figure 1A,B). We performed transcriptome and miRNA sequencing on cells that were experimentally confirmed to be reactivated.

**Figure 2:**
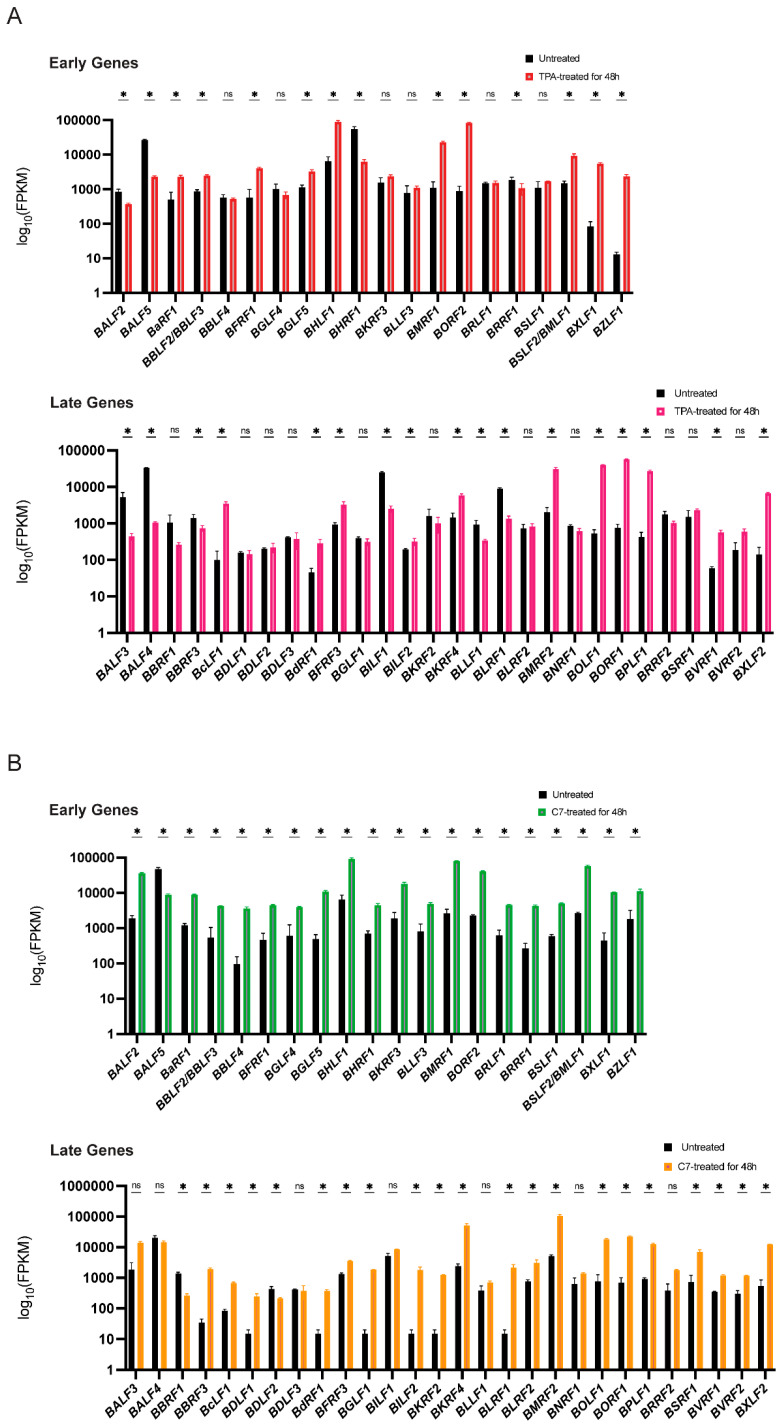
TPA and C7 both induced significant changes in the expression of EBV lytic genes in Raji and C666-1 cells. (**A**) In Raji cells, the TPA treatment resulted in differential expression of early lytic genes (e.g., BZLF1 and BRLF1) and late lytic genes (e.g., BNRF1 and BALF4), as shown by transcriptomic analysis. (**B**) Transcriptomic analysis of C666-1 cells treated with C7 also revealed differential expression of similar EBV lytic genes. * represents a *p*-value < 0.05; ns represents not statistically significant.

Concurrently, mRNA sequencing revealed substantial alterations in the transcriptomes of both Raji and C666-1 cells after 48 h of treatment compared with those of the untreated controls. A distinct subset of viral lytic genes presented increased expression. While both TPA and C7 induced significant changes in gene expression in both cell lines, the specific gene expression profiles differed considerably. These differentially expressed genes suggest a complex interplay between the virus and the host cell during lytic reactivation, with cell line-specific responses to TPA and C7 potentially reflecting differences in cellular signaling pathways and epigenetic regulation (Figure 2A,B).

### 3.2. Differential Expression of miR-7974 During Epstein-Barr Virus (EBV) Reactivation in Raji and C666-1 Cells

**Figure 3:**
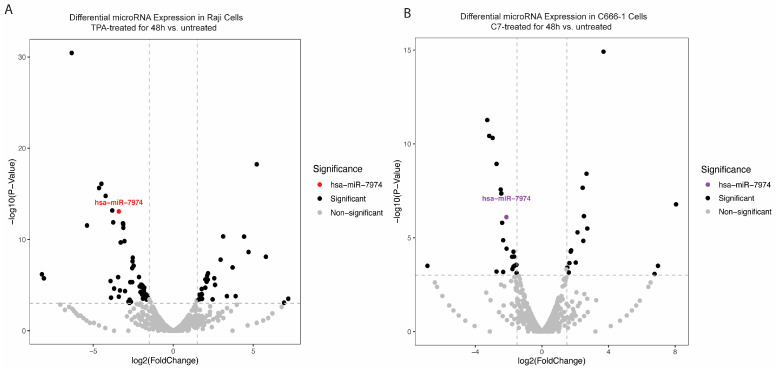
Differential expression of miR-7974 during the reactivation of Raji and C666-1 Cells. (**A**) A volcano plot displaying the statistically significant (*p* < 0.001) results and showing the relationship between the significance of the miRNAs detected and the fold-change between TPA-treated Raji cells at 48 h and untreated Raji cells. miR-7974 was expressed at lower levels in TPA-treated and untreated Raji cells. (**B**) Another volcano plot representing miR-7974 showed a lower abundance in C7-treated and untreated C666-1 cells.

To explore changes in the miRNA expression profile during the reactivation of Raji and C666-1 cells, small RNA sequencing was conducted. The analysis revealed a significant downregulation of miR-7974 in reactivated cells relative to that in untreated controls. Specifically, sequencing reads for miR-7974 were substantially reduced in the reactivated group, with decreases of 3.40-fold in Raji cells (*p* < 0.001) and 2.13-fold in C666-1 cells (*p* < 0.001), indicating a marked reduction in expression (Table 1, Figure 3A,B). The overall distribution of data points in both plots revealed a broad spectrum of miRNAs with altered expression levels post-reactivation. The consistent and significant downregulation of miR-7974 in both cell lines highlights its potential involvement in mediating cellular responses to TPA and C7 stimulation.

**Figure 4:**
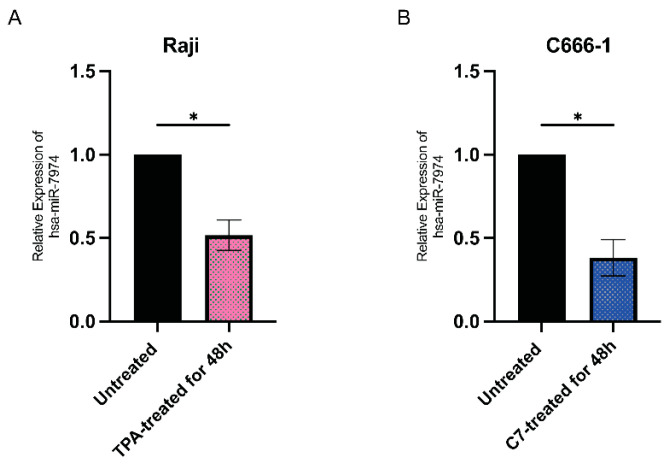
Downregulation of miR-7974 in Raji and C666-1 cells at 48 h post-reactivation. (**A**) Stem-loop reverse transcription quantitative PCR (RT-qPCR) analysis revealed decreased expression of miR-7974 in Raji cells after treatment with TPA. (**B**) RT-qPCR analysis revealed decreased expression of miR-7974 in C666-1 cells after treatment with C7. * Represents a *p*-value < 0.05.

Figure 4 shows the relative expression levels of miR-7974 in two distinct cell lines: Raji cells (Figure 4A) and C666-1 cells (Figure 4B). The quantitative analysis revealed differential expression patterns between the untreated controls (black bars) and the chemically induced groups following 48 h of treatment (colored bars). Figure 4A shows that miR-7974 expression was decreased by 0.48-fold in Raji cells following induction with 20 nM TPA (*p* = 0.012), whereas Figure 4B shows that the miR-7974 expression was decreased by 0.62-fold in C666-1 cells treated with 40 μM C7 (*p* = 0.010). All the data are presented as means ± SDs from three independent experiments and were normalized to those of the untreated controls.

### 3.3. Regulatory Interactions Between miR-7974 and Key Lytic Genes of Epstein-Barr Virus (EBV)

**Figure 5:**
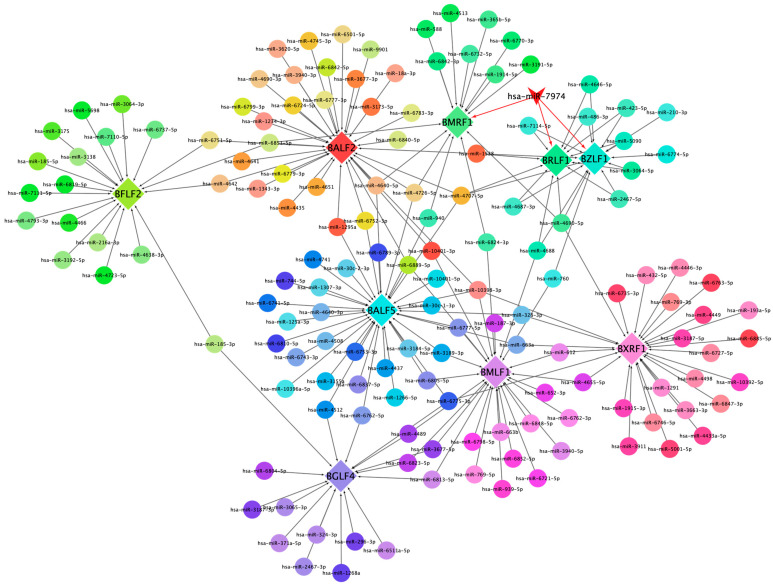
This network diagram illustrates the regulatory interactions between cellular miRNAs and key lytic genes of EBV.

This network diagram illustrates the regulatory interactions between cellular miRNAs and key lytic genes of EBV. The network is visualized with nodes representing both cellular miRNAs and EBV lytic genes, interconnected by edges denoting potential regulatory relationships (Figure 5). Central to the network is miR-7974, prominently highlighted, demonstrating notable connectivity to pivotal EBV lytic genes, including BZLF1, BRLF1, and BMRF1. Specifically, BZLF1 and BRLF1 are critical dual-initiators of the lytic cycle, and encode proteins (ZEBRA and Rta) that trigger the cascade of downstream lytic gene expression. Notably, several miRNAs, including miR-7974, converge on these core regulatory genes, suggesting a potentially significant role in modulating the onset of the lytic cycle. Furthermore, the diagram includes other essential EBV lytic genes, such as BFLF2, BALF5, and BXRF1, which are positioned peripherally but integrated within the network, contributing to its complexity. BALF5 encodes the viral DNA polymerase, which is essential for viral DNA replication; BMRF1 encodes an early antigen involved in viral DNA replication; BXRF1 encodes a viral alkaline exonuclease, which also functions in viral DNA replication; BALF2 participates in viral DNA replication and virion assembly; and BFLF2 contributes to virion assembly and release. The differential coloring of nodes serves to distinguish between individual genes and miRNAs, facilitating clear visualization of specific interactions.

### 3.4. Experimental Validation of miR-7974 Targeting BZLF1 and BRLF1 in Different Human Epithelial Cell Lines

**Figure 6:**
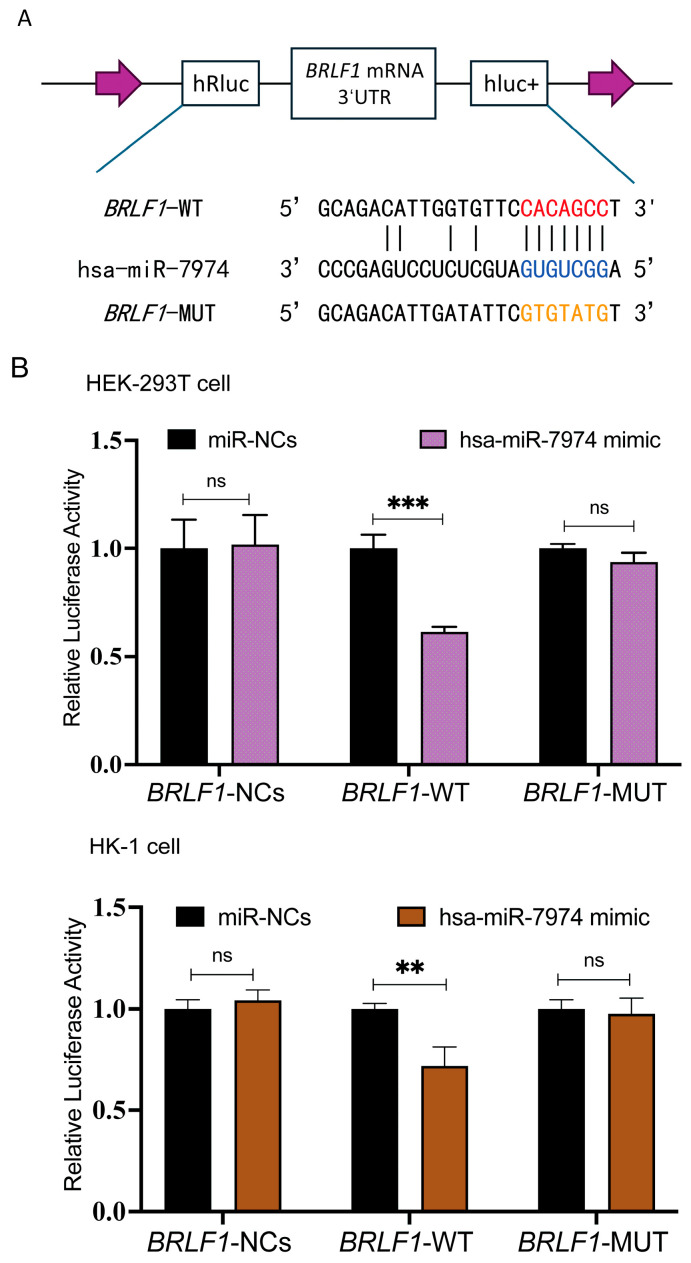
MiR-7974 directly targeted BZLF1 and BRLF1. (**A**) Matching results between the BRLF1 3′ UTR region and the miR-7974 sequence, including the wild type and mutant type of the matching sequences. (**B**) Negative control (BRLF1-NCs), wild type (BRLF1-WT), or mutated (BRLF1-MUT) plasmids were co-transfected with miR-7974 mimics or miR-NCs into HEK293T cells and HK-1 cells. The luciferase activity was measured for 24 h. Statistical analysis was performed with the analysis of variance. *** represents a *p*-value < 0.001; ** represents a *p*-value < 0.01; ns represents not statistically significant.

To investigate whether BZLF1 and BRLF1 represent direct regulatory targets of miR-7974, potential binding sites between miR-7974 and the *3′* UTRs of BZLF1 and BRLF1 were predicted via the miRanda and RNAhybrid online tools. The BZLF1 transcript (ranging from 89,838 to 90,943 in NC_007605.1) entirely overlaps the *3′* UTR sequence of BRLF1 (ranging from 89,838 to 91,077 in NC_007605.1). These analyses suggested a putative miR-7974 binding site within both the BZLF1 *3′* UTR and the BRLF1 *3′* UTR. To further validate this prediction, we generated BRLF1 wild-type (BRLF1-WT) and mutant (BRLF1-MUT) luciferase reporter constructs. The BRLF1-MUT construct contained mutations within the predicted miR-7974 seed region and was designed to disrupt miRNA binding (Figure 6A). Dual-luciferase reporter assays were then performed by co-transfecting HEK293T cells and the EBV-negative human nasopharyngeal carcinoma cell line HK-1 with either a negative control plasmid (BRLF1-NC), the BRLF1-WT reporter, or the BRLF1-MUT reporter, along with either a scrambled miRNA control (miR-NCs) or miR-7974 mimics. Compared with the miR-NC control group, a significant reduction in firefly luciferase activity was observed in cells transfected with the BRLF1-WT reporter, with a 0.61-fold decrease in HEK293T cells (*p* < 0.001) and a 0.72-fold decrease in HK-1 cells (*p* = 0.007). Notably, this inhibitory effect of Hsa-miR-7974 was abolished in both HEK293T and HK-1 cells transfected with the BRLF1-MUT reporter (Figure 6B).

### 3.5. MiR-7974 Suppressed Epstein-Barr Virus (EBV) Reactivation by Significantly Downregulating Epstein-Barr Virus (EBV) Lytic Gene Expression and the Viral Load

**Figure 7:**
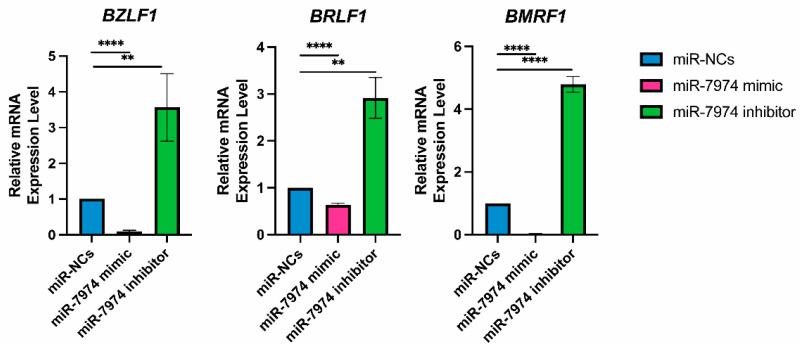
MiR-7974 significantly suppresses EBV lytic gene expression in C666-1 cells. Reverse transcription quantitative PCR (RT-qPCR) was conducted to confirm the significant downregulation of the EBV immediate-early gene BZLF1, BRLF1, and early gene BMRF1, following transfection with the miR-NC (negative control), the miR-7974 mimic, and the miR-7974 inhibitor. **** Represents a *p*-value < 0.0001; ** represents a *p*-value < 0.01.

To analyze the regulatory role of miR-7974 in the expression of EBV immediate-early genes (BZLF1 and BRLF1) and the EBV early gene (BMRF1), the mRNA levels of these genes were screened following transfection of miR-NCs, the miR-7974 mimic, and the miR-7974 inhibitor. The data revealed significant downregulation of BZLF1, BRLF1, and BMRF1 mRNA expression in cells transfected with the miR-7974 mimic compared with the control group (miR-NCs). In contrast, transfection with the miR-7974 inhibitor led to a marked upregulation of all three genes. Specifically, as shown in Figure 7, BZLF1 mRNA expression was decreased by 0.91-fold in the miR-7974 mimic group (*p* < 0.0001) and increased by 3.57-fold in the miR-7974 inhibitor group (*p* = 0.009). Consistent patterns were observed for BRLF1 and BMRF1. BRLF1 mRNA expression decreased by 0.37-fold in the miR-7974 mimic group (*p* < 0.0001) and increased by 2.92-fold in the miR-7974 inhibitor group (*p* = 0.002). Similarly, BMRF1 mRNA expression was decreased by 0.97-fold in the miR-7974 mimic group (*p* < 0.0001) and elevated by 4.79-fold in the miR-7974 inhibitor group (*p* < 0.0001). These results indicate that both genes were significantly downregulated following miR-7974 mimic transfection and significantly upregulated upon miR-7974 inhibitor transfection. These findings indicate that miR-7974 plays a crucial role in regulating the expression of BZLF1, BRLF1, and BMRF1, all of which are essential for EBV lytic cycle initiation and progression.

**Figure 8:**
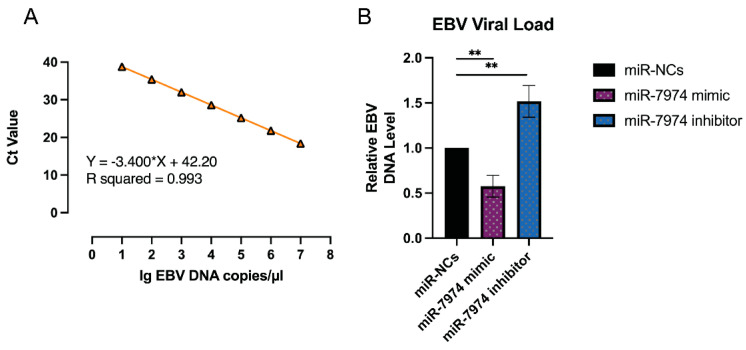
Viral load quantification in response to miR-7974 mimic and inhibitor transfection. (**A**) A standard curve was generated by plotting the cycle threshold (Ct) values against the log of EBV DNA concentrations (R^2^ > 0.99). (**B**) Compared with the miR-NC control group, the miR-7974 mimic group presented a marked reduction in the EBV viral load (*p* = 0.004), whereas the miR-7974 inhibitor group presented a significant increase (*p* = 0.007). ** Represents a *p*-value < 0.01.

The regulation of EBV lytic gene expression by miR-7974, as demonstrated by the significant downregulation of BZLF1, BRLF1, and BMRF1 mRNA levels in C666-1 cells transfected with the miR-7974 mimic, suggests a potential impact on the EBV viral load. To further investigate this, viral load quantification was performed via an EBV probe quantitative PCR kit (Cat# 15-60800, BINGENE, Beijing, China). C666-1 cells transfected with the miR-7974 mimic, the miR-7974 inhibitor, and the negative control (miR-NC) were analyzed to determine the effects of miR-7974 modulation on EBV replication, and 40 ng (2 µL) of each DNA sample was added to carry out each PCR reaction. As shown in Figure 8A, a standard curve was generated by plotting the cycle threshold (Ct) values against the log of DNA concentrations (from 10^7^ copies/µL to 10 copies/µL). The viral load in cells transfected with the miR-7974 mimic was significantly reduced compared to the miR-NC group (0.58-fold, *p* = 0.004), which was consistent with the observed downregulation of the BZLF1, BRLF1, and BMRF1 mRNA levels. These genes are critical for the initiation and progression of the EBV lytic cycle, and their suppression likely hinders viral replication, leading to a lower viral load. Conversely, transfection with the miR-7974 inhibitor resulted in a significant increase in the viral load (1.52-fold, *p* = 0.007), and this upregulation likely promoted the EBV lytic cycle and increased viral replication, thereby increasing the overall viral load (Figure 8B). On the basis of the standard curve quantification, the median viral load following C7 activation was determined to be 6640 copies/ng DNA in cells transfected with miR-NC, 3029 copies/ng DNA in cells transfected with the miR-7974 mimic, and 8744 copies/ng DNA in cells transfected with the miR-7974 inhibitor.

### 3.6. Significantly Enriched Signaling Pathways, Disease, and Functional Clustering of Target Genes of Validated Targets of miR-7974

**Figure 9:**
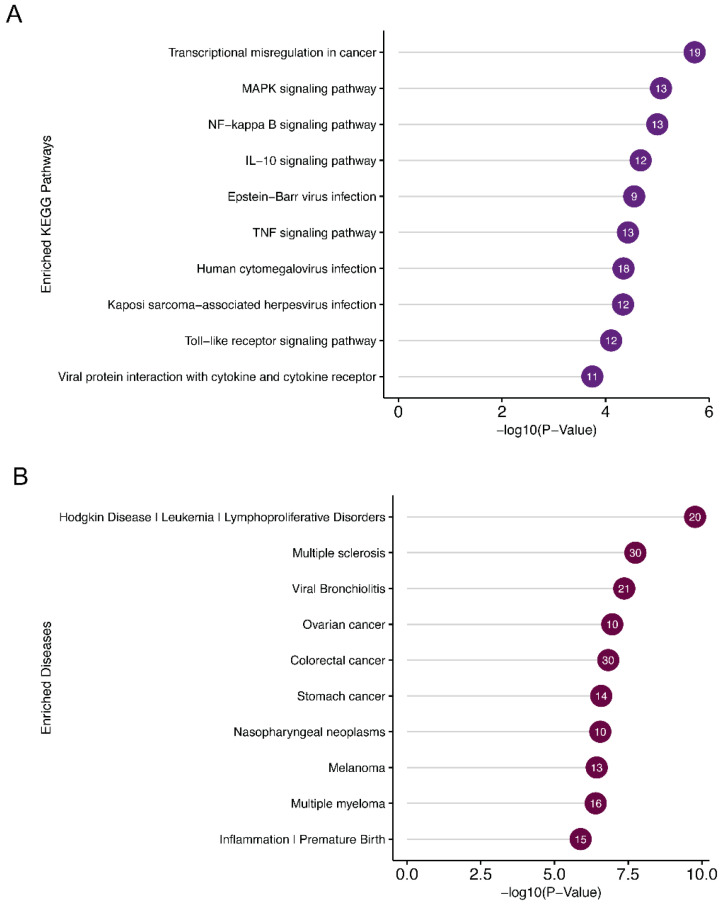

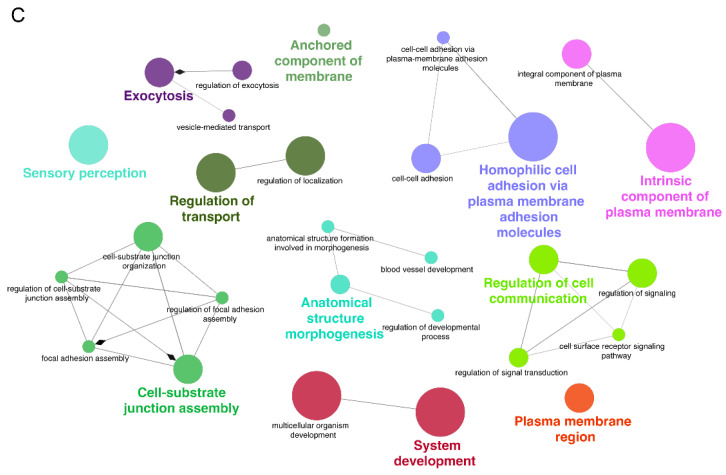
Pathway and disease enrichment analysis of miR-7974 target genes, along with the functional cluster network of these targets. (**A**) Lollipop plots show the significantly enriched signaling pathways of the validated targets of miR-7974. (**B**) Lollipop plots show the significantly enriched diseases associated with the validated targets of miR-7974. (**C**) The GO enrichment analysis network shows the functional clustering of target genes of miR-7974 via the ClueGO and CluePedia plugins in the Cytoscape software 3.10.3.

DAVID functional annotation of the miR-7974 target genes revealed significant enrichment of several KEGG pathways and disease associations related to EBV reactivation (Figure 9A,B). Notable KEGG pathways included the “MAPK signaling pathway”, “NF-κB signaling pathway”, and “IL-10 signaling pathway”, all of which play crucial roles in regulating EBV latency and lytic cycle transition. Specifically, the NF-κB pathway is known to be a key mediator of EBV reactivation, and its modulation by miR-7974 may directly influence the virus’s ability to switch from latency to lytic replication. Furthermore, the “Epstein-Barr virus infection” pathway itself was significantly enriched, highlighting the direct involvement of miR-7974 target genes in viral processes. Disease association analysis identified “nasopharyngeal neoplasms” as a prominent hit, which is consistent with the established link between EBV and this malignancy. Additionally, “lymphoproliferative disorders” and “leukemia” were significantly enriched, reflecting the broader impact of EBV on hematological malignancies. The enrichment of “Inflammation” and “Premature Birth” also suggests potential roles for miR-7974 in modulating inflammatory responses and reproductive health, which may indirectly influence EBV reactivation or its associated pathologies.

GO enrichment analysis of the miR-7974 target genes, which was conducted and visualized through the ClueGO plugin within Cytoscape (Figure 9C), revealed significant enrichment in biological processes closely linked to membrane dynamics, cellular adhesion, and signal transduction—key pathways implicated in EBV reactivation. Specifically, terms such as “anchored component of membrane” (GO:0031225), “integral component of plasma membrane” (GO:0005887), and “plasma membrane region” (GO:0098590) were prominently enriched, suggesting that miR-7974 may regulate EBV reactivation by modulating genes involved in the processing and assembly of viral envelope glycoproteins, which are critical for virion release. The analysis further revealed significant enrichment of cell adhesion-related processes, particularly “cell-cell adhesion via plasma membrane adhesion molecules” (GO:0098742) and “homophilic cell adhesion via plasma membrane adhesion molecules” (GO:0007156), implicating miR-7974 in the regulation of intercellular viral dissemination mechanisms critical for EBV pathogenesis. Notably, significant enrichment was also identified in functional categories such as “regulation of transport” (GO:0051049), “regulation of cell communication” (GO:0010646), and “regulation of signal transduction” (GO:0023052), suggesting that miR-7974 has the potential to modulate EBV lytic cycle progression through host cell signaling networks. Furthermore, the enrichment of terms such as “anatomical structure morphogenesis” (GO:0009653) and “system development” (GO:0048731), as visualized through ClueGO network analysis, suggests broader implications for miR-7974 in EBV-associated oncogenesis. Together, these findings, derived from a comprehensive network-based GO enrichment analysis, highlight the pleiotropic regulatory role of miR-7974 in EBV biology and provide valuable insights into novel therapeutic targets for EBV-associated diseases.

## 4. Discussion

EBV, a ubiquitous human gammaherpesvirus, establishes lifelong latent infection in more than 90% of the global adult population. Its ability to switch between latent and lytic phases is crucial for viral persistence and pathogenesis, particularly in EBV-associated malignancies such as nasopharyngeal carcinoma, Burkitt’s lymphoma, and Hodgkin’s lymphoma. The transition from latency to lytic replication is tightly regulated by both viral and host factors, including miRNAs. MiRNAs are small non-coding RNAs that play pivotal roles in post-transcriptional gene regulation, often by binding to the 3′ UTR of target mRNAs, leading to mRNA degradation or translational repression. While EBV-encoded miRNAs have been extensively studied, the role of host miRNAs in regulating EBV reactivation remains poorly understood. A study by Cramer et al. (2014) demonstrated that miR-190 promotes host cell survival and prevents EBV from entering the lytic life cycle [21]. In this study, we identified miR-7974 as a novel regulator of EBV reactivation, demonstrating its ability to directly target and suppress the expression of two key viral lytic genes, *BZLF1* and *BRLF1*.

Multiple pharmacological agents, including romidepsin [29], NEO-212 [30], valproic acid [31], and suberoylanilide hydroxamic acid (SAHA) [32], have been reported to effectively induce lytic reactivation in C666-1 cells. Notably, the choice of lytic inducer may influence experimental outcomes, as different agents can activate distinct signaling pathways. Whether downregulation of miR-7974 is a conserved feature of EBV solubility reactivation or a drug-specific artifact is a question worth considering. Therefore, when we designed the protocol for this study, we did consider the drug-specific effects. We employed two cell model systems: Raji cells treated with TPA, and C666-1 cells treated with C7. In both systems, successful EBV lytic replication was induced, accompanied by significant reduction in miR-7974 expression (as shown in Table 1 and Figure 3 and Figure 4). This suggested that the changes in miR-7974 expression in EBV-infected Raji cells are attributable primarily to viral reactivation rather than TPA-mediated cellular effects. Thus, we can similarly assume that the situation in C666-1 cells is also consistent. And, therefore, we suggest that downregulation of miR-7974 is a conserved feature of EBV lytic reactivation rather than a drug-specific artifact. In addition, C7 has been extensively documented as an effective lytic inducer in epithelial-derived cell lines, including NPC models [33,34]. Our preliminary experiments also confirmed that C7 induced a consistent and reproducible lytic cycle response in C666-1 cells, as measured by the expression of early lytic genes and viral genome replication; therefore, we selected C7 as the primary lytic inducer for C666-1 cells.

We have identified miR-7974 as a critical regulator of EBV reactivation, demonstrating its ability to directly target and suppress the expression of two pivotal viral lytic genes, *BZLF1* and *BRLF1*. We observed a significant downregulation of *BZLF1* and *BRLF1* following transfection with miR-7974 mimics, and a corresponding upregulation upon miR-7974 inhibition. The direct interaction between miR-7974 and the 3′ UTR of *BRLF1* was further validated via dual-luciferase reporter assays, and the downregulation of the mRNA expression of the *BZLF1* and *BRLF1* genes following miR-7974 transfection provided robust mechanistic evidence for this regulatory relationship. These findings further support the role of miR-7974 as a negative regulator of EBV lytic gene expression and viral replication. The inverse correlation between the miR-7974 level and viral load underscores the potential of miR-7974 as a therapeutic target for controlling EBV replication and associated diseases. Zhang et al. (2010) reported that Hsa-199a-3p and Hsa-210-3p suppress hepatitis B virus (HBV) replication by targeting the HBV pre-S1 region [35], similar to the mechanism of action of miR-7974. Future studies should explore the mechanisms by which miR-7974 modulates viral gene expression and its broader implications for EBV latency and reactivation.

Our results align with and extend previous findings on the regulatory roles of miRNAs in the EBV lytic-latent switch. EBV reactivation is precisely regulated by an intricate crosstalk network of viral and host miRNAs [9]. For example, EBV-encoded miR-BARTs have been shown to repress *BZLF1* and *BRLF1* expression, thereby promoting viral latency. Lung et al. (2018) suggested that BART5-5p, BART7-3p, BART9-3p, and BART14-3p control the ATM signaling pathway in NPC cells. By suppressing these endogenous miR-BARTs in EBV-positive NPC cells, a novel function of miR-BARTs in inhibiting Zta-induced lytic reactivation was discovered [36]. Jung et al. (2014) identified miR-BART20-5p as a potent regulator of EBV latency through specific targeting of the *BRLF1* 3′ UTR, effectively blocking viral reactivation [18]. Barth et al. (2008) identified miR-BART2-mediated suppression of *BALF5* (viral DNA polymerase), with miRNA overexpression achieving 40–50% BALF5 protein reduction and 20% decrease in virion production, effectively maintaining viral latency [37]. Ellis-Connell et al. (2010) reported that miR-200b and miR-429 serve as lytic inducers that counteract ZEB1/ZEB2-mediated repression of the *BZLF1* Zp promoter across EBV-infected cell types [38]. Chen et al. (2021) identified miR-142 as a critical regulator that suppresses EBV reactivation by modulating the BCR-initiated SOS1/Ras/Raf/Mek/Erk signaling cascade [20]. Cao et al. (2018) demonstrated that ultraviolet (UV) irradiation and hypoxia induce EBV reactivation in lymphoma cells, and miR-18a serves as a key mediator which promotes EBV reactivation by suppressing the ATM-dependent DNA damage response (DDR) pathway [39]. However, in contrast to these previously characterized miRNAs, miR-7974 has been implicated in various cancer contexts. Its role in EBV biology, as revealed in this study, adds a novel dimension to its functional repertoire and highlights its potential as a key player in viral–host interactions. The functional role of miR-7974 in cancer appears to be highly context-dependent. While some studies have reported its upregulation in breast and colorectal cancers, suggesting a potential oncogenic function, others have reported its downregulation in glioma, where it acts as a tumor suppressor by targeting oncogenes such as *CORO2A*, *APC2*, *WNT7A*, and *BOC* [27]. This duality underscores the tissue-specific and context-dependent nature of the miRNA function, which may be influenced by the unique molecular and cellular environments of different cancer types.

Comprehensive bioinformatics analyses (GO enrichment, KEGG pathway, and disease association analyses) revealed the broader regulatory network of miR-7974. GO analysis revealed significant enrichment in biological processes related to membrane dynamics, cellular adhesion, system development, and signal transduction—key pathways critically implicated in EBV reactivation. These findings suggest that miR-7974 may regulate EBV reactivation not only through direct viral gene targeting, but also by modulating host genes involved in viral envelope glycoprotein processing, assembly, and intercellular viral dissemination. Zheng et al. (2023) reported that the miR-7974 inhibitor reduced the osteogenic differentiation of dental follicle stem cells by targeting *FKBP15*, potentially impacting skeletal development [40]. According to the research by Kumar et al. (2020), miR-7974 was predicted to regulate several gene clusters (*NOG*, *S1PR1*, *MRAS*, *PDGFC,* and *BMP5*) involved in early neural induction of human embryonic stem cells and induced pluripotent stem cells [41]. KEGG analysis highlighted the enrichment of key signaling pathways, including the “MAPK signaling pathway”, “NF-κB signaling pathway”, and “IL-10 signaling pathway”, all of which are pivotal in regulating EBV latency and lytic cycle transitions. Notably, disease association analysis identified “nasopharyngeal neoplasms”, “lymphoproliferative disorders”, and “leukemia” as prominent hits, reflecting the well-established links between EBV and these malignancies. According to the research by Gao et al. (2019), the PKC, NF-κB, p38 MAPK, PI3K, and ERK signaling pathways have been implicated in EBV reactivation [42]. Nanbo et al. (2012) reported that ERK and NF-κB pathway inhibitors effectively suppressed both lytic induction and cell-to-cell EBV transmission [43]. Chen et al. (2023) reported that miR-7974 can regulate the immune processes by modulating several pathways, such as the mTOR and PI3K-Akt signaling pathway [44]. Dong et al. (2020) reported that the pathway enrichment analyses of the miR-7974 target genes were partly associated with the cytokine–cytokine receptor interaction [45]. These findings are generally consistent with the miR-7974-related pathways identified in this study.

Several critical questions remain to be addressed in future studies. First, the precise molecular mechanisms by which miR-7974 regulates *BZLF1* and *BRLF1* expression, including the identification of specific binding sites and the involvement of RNA-induced silencing complex (RISC) components, warrant further investigation. Second, the impact of miR-7974 on other EBV lytic genes and the overall viral life cycle needs to be systematically explored. Third, the clinical relevance of miR-7974 expression levels in EBV-associated malignancies and their correlation with disease progression and patient outcomes should be investigated to validate its potential as a biomarker or therapeutic target.

Our study has certain limitations. While we have demonstrated the direct targeting of *BZLF1* and *BRLF1* by miR-7974, the broader impact of miR-7974 on host cellular pathways that may indirectly influence EBV reactivation remains to be fully elucidated. Additionally, further research is needed to explore the effects of miR-7974 on the protein levels of ZEBRA and Rta, as well as the downstream consequences on viral replication and particle production. Future studies should also investigate the interplay between miR-7974 and other miRNAs or host factors that may synergistically regulate EBV reactivation.

## 5. Conclusions

This study revealed that miR-7974 functions as a critical regulator of EBV reactivation by directly targeting the immediate-early genes *BZLF1* and *BRLF1*. The significant downregulation of miR-7974 during EBV reactivation, coupled with its ability to suppress lytic gene expression, highlights its role as a negative regulator of the viral lytic cycle. Functional enrichment analyses via GO, KEGG pathway, and disease association analyses, advance our understanding of the complex interplay between host miRNAs and EBV. These findings suggest that miR-7974 may serve as a potential therapeutic target for EBV-associated malignancies, offering new insights into the molecular mechanisms underlying EBV reactivation and its associated diseases. Further research into the molecular mechanisms and clinical applications of miR-7974 modulation may pave the way for novel therapeutic strategies in the management of EBV-associated malignancies, offering hope for improved outcomes in patients with these challenging diseases.

## Figures and Tables

**Table 1:** MiR-7974 was differentially expressed in TPA-treated Raji cells and C7-treated C666-1 cells compared with untreated controls.

Cell Line	miRNA	Expression	*p*-Value (Treated vs. Untreated Cells)	P-Adjust (Treated vs. Untreated Cells)	log2(FoldChange)
Raji cell	miR-7974	Downregulated	8.46 × 10^−14^	1.45 × 10^−11^	−3.399
C666-1 cell	miR-7974	Downregulated	7.98 × 10^−7^	1.03 × 10^−4^	−2.132

## Data Availability

The original contributions presented in the study are included in the article. Further inquiries can be directed to the corresponding author or the first author.
